# Controlling the Spatial Spread of a Xylella Epidemic

**DOI:** 10.1007/s11538-021-00861-z

**Published:** 2021-02-17

**Authors:** Sebastian Aniţa, Vincenzo Capasso, Simone Scacchi

**Affiliations:** 1 Faculty of Mathematics, “Alexandru Ioan Cuza” University of Iaşi, 700506 Iaşi, Romania; 2 “Octav Mayer” Institute of Mathematics of the Romanian Academy, 700506 Iaşi, Romania; 3 ADAMSS (Centre for Advanced Applied Mathematical and Statistical Sciences), Universitá degli Studi di Milano “La Statale”, 20133 Milan, Italy; 4 Department of Mathematics, Universitá degli Studi di Milano “La Statale”, 20133 Milan, Italy

**Keywords:** *Xylella fastidiosa*, Olive trees, Epidemics, Mathematical model, Reaction–diffusion models, Numerical simulations, Control strategies, Regional control, 35-XX, 35B40, 37N25, 92C80, 92D30, 92D40, 93B99

## Abstract

In a recent paper by one of the authors and collaborators, motivated by the Olive Quick Decline Syndrome (OQDS) outbreak, which has been ongoing in Southern Italy since 2013, a simple epidemiological model describing this epidemic was presented. Beside the bacterium *Xylella fastidiosa*, the main players considered in the model are its insect vectors, *Philaenus spumarius*, and the host plants (olive trees and weeds) of the insects and of the bacterium. The model was based on a system of ordinary differential equations, the analysis of which provided interesting results about possible equilibria of the epidemic system and guidelines for its numerical simulations. Although the model presented there was mathematically rather simplified, its analysis has highlighted threshold parameters that could be the target of control strategies within an integrated pest management framework, not requiring the removal of the productive resource represented by the olive trees. Indeed, numerical simulations support the outcomes of the mathematical analysis, according to which the removal of a suitable amount of weed biomass (reservoir of *Xylella fastidiosa*) from olive orchards and surrounding areas resulted in the most efficient strategy to control the spread of the OQDS. In addition, as expected, the adoption of more resistant olive tree cultivars has been shown to be a good strategy, though less cost-effective, in controlling the pathogen. In this paper for a more realistic description and a clearer interpretation of the proposed control measures, a spatial structure of the epidemic system has been included, but, in order to keep mathematical technicalities to a minimum, only two players have been described in a dynamical way, trees and insects, while the weed biomass is taken to be a given quantity. The control measures have been introduced only on a subregion of the whole habitat, in order to contain costs of intervention. We show that such a practice can lead to the eradication of an epidemic outbreak. Numerical simulations confirm both the results of the previous paper and the theoretical results of the model with a spatial structure, though subject to regional control only.

## Introduction

The etiological agent of olive tree disease known as olive quick decline syndrome (OQDS) is the plant pathogenic bacterium *Xylella fastidiosa*, which is a vector-borne bacterium.

The main vector of *Xylella fastidiosa* in Southern Italy has been identified in the so-called meadow spittlebug, i.e., the Philaenus spumarius, a xylem sap-feeding specialist. Their juvenile form (nymphs) develops on weeds or ornamental plants, confined in a foam produced by themselves for protection from predators and temperature, while their adult form moves to olive tree canopies. Experiments have shown a larger infection prevalence of adults on olive trees than on weeds; this fact may lead to the assumption of infection of adults from infected olive trees more than from weeds.

*X. fastidiosa* transmission is the result of four events [see e. g. Almeida et al. (2005), Redak et al. (2004) and references cited therein]:

(a) acquisition from a source plant;

(b) attachment and retention to the vector’s foregut;

(c) inoculation into a new host;

(d) development of the infection in a plant after inoculation.

A successful transmission also includes bacterial multiplication.

Once a plant is infected, bacteria multiply within the xylem vessels inducing the production of a gel in the plant xylem, which occludes the xylem vessels, thus inhibiting the flux of water through the lymph vessels eventually blocking the nutrition of the plant. Typical symptoms are leaf scorch, dieback of twigs, branches and even of the whole plant (Carlucci et al. 2013).

Sanitation of infected olive trees is unfeasible; the scope of our research is the mathematical modeling of the population dynamics of the ecosystem in the presence of infection. The availability of a sound mathematical model may lead to predictive analysis of the relevant populations, so as to suggest possible eradication strategies, or at least possible optimal control strategies.

In a previous paper (Brunetti et al. 2020), based on the outbreak of OQDS in Southern Italy, a model describing the epidemic was presented. It consists of a system of ordinary differential equations (ODEs) describing the evolution of the main three players, i.e., the insect vector, *Philaenus spumarius*, the olive trees, and weeds. A preliminary mathematical analysis and related numerical simulations have shown that “the removal of a significant amount of weeds (acting as a reservoir for juvenile insects) from olive orchards and surrounding areas has resulted in the most efficient strategy to control the spread of the OQDS. In addition, as expected, the adoption of more resistant olive tree cultivars has been shown to be a good strategy, though less cost effective, in controlling the pathogen.”

The scope of the present paper is to extend the above results to a spatially structured model and to show in a more rigorous way that the spatial expansion of an OQDS outbreak can indeed be stopped by acting either on the weed biomass or on the choice of the olive cultivar. We have adopted a deterministic reaction–diffusion model. We recall that reaction–diffusion models can be interpreted as mean-field approximations of individual based models, which are more appropriate at the micro scale. Of course, in our case individual behaviors and possible randomness are lost. On the other hand, our approach allows a mathematical “qualitative” analysis of the system, including the derivation of eradicability theorems. Such a “qualitative” analysis has driven on the one side, our numerical experiments and, on the other side, anticipates future investigations on optimal control problems in a variety of scenarios.

In order to keep mathematical technicalities to a minimum, here the weed biomass is taken as a given field parameter and as in Brunetti et al. (2020), the insect life cycle has not been taken into account [more information about this last aspect can be found in Rossini et al. (2020)]. As possible control actions, insect traps, weed cut, choice of the cultivar, and reduction of contact rates have been taken into account. In the numerical simulations, particular attention has been paid to weed cut and choice of the cultivar, the other control measures being more difficult to implement. It is worth mentioning that, in recent investigations presented in Schneider et al. (2020), the authors, by means of a cellular automaton simulator, have confirmed the relevance of the olive cultivar as a possible effective control strategy.

A relevant contribution of our approach consists of the restriction of measures of intervention (control) only to a subregion of the whole habitat of interest. Due to diffusion, any point of the domain $\varOmega$ is strongly connected to any other point of $\varOmega ,$ so that any action taken in a subregion $\omega \subset \varOmega$ will eventually propagate to the whole habitat. This is the “leit motiv” of our proposal concerning regional control: “think globally, act locally” [see Aniţa and Capasso (2009)]. This practice may contribute in a significant way to improve the ratio cost/effectiveness of real control strategies. Mathematical analysis and numerical simulations have been carried out showing that it is indeed possible to eradicate the disease by such local action. Our aim is to analyze optimal control problems in future investigations, which may possibly lead to the identification of an optimal choice of the subregion of intervention.

The paper is organized as follows. In Sect. 2, the mathematical model is presented. In Sect. 3, possible control strategies are proposed, based on which an eradicability result is shown. Finally, in Sect. 4, numerical simulations are presented which confirm the analytical results. In the numerical simulations, the relevant parameters have been taken from Brunetti et al. (2020).

## Our Model

Since the feeding behavior and metabolic processes are qualitatively similar for both nymphs and adults (Janse and Obradovic 2010), we will consider only one stage of active vectors of the infection. We will consider a spatially structured model which includes the population of vector insects and the population of infected olive trees.

We will denote by $C_I(x, t)$ the spatial density of the total population of insects, by $s_1(x, t)$ the spatial density of susceptible insects, and by $i_1(x, t)$ the spatial density of infected insects:

$$\begin{aligned} C_I(x, t) = s_1(x, t) + i_1(x, t). \end{aligned}$$

$C_T(x, t)$ will denote the spatial density of the total biomass of olive trees, $s_2(x, t)$ the spatial density of the biomass of healthy trees, $i_2(x, t)$ the spatial density of the biomass of infected trees:

$$\begin{aligned} C_T(x, t) = s_2(x, t) + i_2(x, t). \end{aligned}$$

All the parameters in the model are nonnegative quantities.

- INSECTS

Due to the fact that bacteria reside only in the foregut of an adult insect, the latter generate only healthy offspring [see, e.g., Almeida et al. (2005), Redak et al. (2004), and references cited therein]. The parameter *r* denotes the birth rate of new insects.

Reproduction, however, can occur only if bushes and other plants, whether healthy or infected, are present, explaining the presence of a “carrying capacity” *M*(*x*) in the logistic term, describing the biomass of all such plants, which we simply call *weeds*.

Both healthy and infected insects experience natural mortality at a rate *n*;  $\chi$ is a scale parameter.

Finally, we assume that insects may diffuse in the habitat (with constant diffusion coefficient to avoid purely technical complications).

As far as the local incidence of infection for insects, at point $x\in \overline{\varOmega }$, and time $t\ge 0$, as in previous models (Capasso 1984; Aniţa and Capasso 2009), we assume that it arises due to biting of infected olive trees at any point $x^{\prime }\in \varOmega$ of the habitat, within a spatial neighborhood of *x* represented by a suitable probability kernel $k(x,x^{\prime }),$ depending on the specific structure of the local ecosystem [see also Shcherbacheva et al. (2018)]; as a trivial simplification, one may assume $k(x, \cdot )$ as a Gaussian density centered at *x*;  hence, the “local incidence rate (i.r.)” for insects, at point $x\in {\ \varOmega }$, and time $t\ge 0$, is taken as

$$\begin{aligned} (i.r.)_I(x,t)= \beta s_1 (x, t)\int _{\varOmega }k(x,x^{\prime }) i_2(x^{\prime },t)\mathrm{d}x^{\prime }. \end{aligned}$$

Hence, the spatial dynamics of susceptible insects is expressed by the following equation

1 $$\begin{aligned} \frac{\partial s_1}{\partial t} (x, t)&= d\varDelta s_1(x, t) + r C_I(x, t) [M(x) - \chi s_1(x, t)] -n s_1(x, t) \nonumber \\&\quad -\, \beta s_1(x, t) \displaystyle \int _{\varOmega }k(x,x^{\prime })i_{2}(x^{\prime },t)\mathrm{d}x^{\prime }, \end{aligned}$$

while the spatial dynamics of infective insects is expressed by the following equation

2 $$\begin{aligned} \frac{\partial i_1}{\partial t}(x,t)&=d\varDelta i_{1}(x,t)\displaystyle - n i_{1}(x,t) - r \chi i_{1}(x,t) C_I(x, t) \nonumber \\&\quad + \, \beta s_1(x, t) \displaystyle \int _{\varOmega }k(x,x^{\prime })i_{2}(x^{\prime },t)\mathrm{d}x^{\prime }. \end{aligned}$$

Both Eqs. (1) and (2) act in a spatial domain $\varOmega \subset \mathbb {R}^2,$ at times $t \in (0,+\infty ).$ They are subject to suitable initial conditions, and we assume homogeneous Neumann boundary conditions.

- OLIVE TREES

For the olive trees, it is better to refer to their canopies, so that we may consider pruning and regrowth. Healthy trees (canopy) are produced at constant net regrowth rate *q*,  get infected by contact with infected insects at rate $\zeta$ or by human activities, such as budding and grafting, at rate *b*. For trees, in view of their long survival, we can neglect natural mortality. Infected trees experience disease-related mortality $\mu$ and human-induced mortality $\ell$ due to pruning and logging.

Hence, the spatial dynamics of trees is expressed by the following equations

3 $$\begin{aligned} \frac{\partial s_2}{\partial t} (x, t)&= (q-\ell ) s_2(x, t) - s_2(x, t) \displaystyle \frac{C_T(x, t)}{C} \nonumber \\&\quad - (\zeta i_1(x, t) + b \ell i_2(x, t)) s_2(x, t) + \alpha i_2(x, t), \end{aligned}$$

4 $$\begin{aligned} \frac{\partial i_2}{\partial t} (x, t)&= - \mu i_2(x, t) - \ell i_2 (x, t)- i_2(x, t) \displaystyle \frac{C_T(x, t)}{C} \nonumber \\&\quad + (\zeta i_1(x, t) + b \ell i_2(x, t)) s_2(x, t) - \alpha i_2(x, t). \end{aligned}$$

Both Eqs. (3) and (4) act in the spatial domain $\varOmega \subset \mathbb {R}^2,$ at times $t \in (0,+\infty ).$ They are subject to suitable initial conditions.

**Assumptions:**

- $\varOmega \subset \mathbb {R}^2$ is a bounded domain with a smooth boundary;
- $$M\in L^{\infty }(\varOmega )$$, $$M(x)\ge 0$$ a.e. in $$\varOmega $$;
- $k\in L^{\infty }(\varOmega \times \varOmega )$, $k(x,x')\ge 0$ a.e. in $\varOmega \times \varOmega$; normalized to $\beta ;$
- $d, \ r, \ \chi , n, \ q, \ \ell , \ C, \ \mu , \ \zeta , \ b , \ \alpha , \ \beta$ are positive constants.

**Table 1:** Model parameters

Symbol	Description
*r*	Insect birth rate
$\chi$	Insect intraspecific competition rate
*n*	Insect mortality rate
*q*	Healthy tree (canopy) regrowth rate
*C*	Tree carrying capacity parameter
$\ell$	Elimination rate of trees by pruning or logging
*b*	Infection rate of trees by infected tools
$\mu$	Infected tree mortality rate
$\alpha$	Infected tree recovery rate
$\beta$	Insect infection rate by infected trees
$\zeta$	Tree infection rate by infected insects

## Control and Eradicability

By summing Eqs. (1) and (2), we obtain an equation for the total insect population $C_I(x, t)$

$$\begin{aligned} \frac{\partial C_I}{\partial t} (x, t) = d\varDelta C_I (x, t) + r C_I(x, t) [M(x) - \chi C_I(x, t)] -n C_I(x,t). \end{aligned}$$

For *M* constant, pure Neumann boundary conditions allow constant in space and time solutions, satisfying the following equilibrium equation

$$\begin{aligned} r C_I^* (M - \chi C_I^*) -nC_I^*=0, \end{aligned}$$

that we may rewrite as

$$\begin{aligned} {[}r (M - \chi C_I^*) -n] C_I^* =0. \end{aligned}$$

This equation may admit two solutions

$$\begin{aligned} C_I^* =0, \end{aligned}$$

and the solution of

$$\begin{aligned} r (M - \chi C_I^{**}) -n =0. \end{aligned}$$

This shows that for $M\le \displaystyle {n\over r}$, no other nonnegative solutions are feasible, but the trivial one. Otherwise, another equilibrium is feasible

5 $$\begin{aligned} C_I^{**} = \frac{1}{\chi } \left( M - \frac{n}{r} \right) , \end{aligned}$$

provided

$$\begin{aligned} M > \frac{n}{r}. \end{aligned}$$

From the above simple reasoning, we may conjecture that a way to eradicate the disease is to eliminate the insect population by a significant reduction of the carrying capacity *M*,  which may be obtained by eliminating weeds in the relevant olive orchards. Equation (5) shows the quantitative role of the scale parameter $$\chi ;$$ a smaller value of $$\chi $$ allows a larger value of $$C_I^{**},$$ so that we may say that $$\displaystyle {\frac{M}{\chi }}$$ plays the role of an “effective carrying capacity” of insects. Numerical simulations, reported in Sect. 4, illustrate these facts.

A more accurate analysis follows. Assume that certain constant controls are considered in a non-empty open subset $\omega \subset \varOmega$.

Possible regional controls (acting in $\omega$) are the following:

1. Traps : $n \rightarrow n + \gamma _1;$ increase insect death rate by insecticides and/or treated nets.
2. Weed cut : $M \rightarrow M(1 - \gamma _{21});$ decrease carrying capacity of insects by eliminating weeds in the olive orchards.
3. Choice of the cultivar: $\zeta \rightarrow \zeta (1 - \gamma _{22});$ decrease infection rate of trees, acting on the cultivar.
4. Reduce contacts: $\beta \rightarrow \beta (1 - \gamma _{23}) ;$ decrease contact rate of trees with insects by installing treated nets.
5. Clean tools: $b \rightarrow b(1 - \gamma _3);$ decrease infection rate from tools to trees by disinfection.

For various reasons, including cost reduction, we may consider the possibility of implementing the proposed control measures only on a suitable subregion $\omega \subset \varOmega$ of the whole habitat, including areas already affected by the epidemic, augmented by a preventive confinement area.

Let $\mathbb I_{\omega }$ denote the characteristic function of $\omega$. Then, the controlled system is

6 $$\begin{aligned} \frac{\partial s_1}{\partial t} (x, t)&= d\varDelta s_1(x, t) + r C_I(x, t) [ M(x)(1 - \gamma _{21}\mathbb I_{\omega }(x)) - \chi s_1(x, t)] \nonumber \\&\quad - (n + \gamma _1\mathbb I_{\omega }(x)) s_1(x, t) - \beta (1 - \gamma _{23}\mathbb I_{\omega }(x)) s_1(x, t) \displaystyle \int _{\varOmega }k(x,x^{\prime })i_{2}(x^{\prime },t)\mathrm{d}x^{\prime }, \end{aligned}$$

7 $$\begin{aligned} \frac{\partial i_1}{\partial t}(x,t)&=d\varDelta i_{1}(x,t)\displaystyle - (n + \gamma _1\mathbb I_{\omega }(x)) i_{1}(x,t) - r \chi i_{1}(x,t) C_I(x, t) \nonumber \\&\quad + \beta (1 - \gamma _{23}\mathbb I_{\omega }(x)) s_1(x, t) \displaystyle \int _{\varOmega }k(x,x^{\prime })i_{2}(x^{\prime },t)\mathrm{d}x^{\prime }, \end{aligned}$$

8 $$\begin{aligned} \frac{\partial s_2}{\partial t} (x, t)&=(q-\ell ) s_2(x, t) - s_2(x, t) \displaystyle \frac{C_T(x, t)}{C} \nonumber \\&\quad - (\zeta (1- \gamma _{22}\mathbb I_{\omega }(x)) i_1(x, t) + b(1 - \gamma _3\mathbb I_{\omega }(x)) \ell i_2(x, t)) s_2(x, t) + \alpha i_2(x, t), \end{aligned}$$

9 $$\begin{aligned} \frac{\partial i_2}{\partial t} (x, t)&= - \mu i_2(x, t) - \ell i_2 (x, t)- i_2(x, t) \displaystyle \frac{C_T(x, t)}{C} \nonumber \\&\quad + (\zeta (1- \gamma _{22}\mathbb I_{\omega }(x)) i_1(x, t) + b(1 - \gamma _3\mathbb I_{\omega }(x)) \ell i_2(x, t)) s_2(x, t) - \alpha i_2(x, t). \end{aligned}$$

We assume that there is no flux of insects through the boundary of $\varOmega$ (the domain is isolated):

10 $$\begin{aligned} \frac{\partial s_1}{\partial \nu } (x, t)=\frac{\partial i_1}{\partial \nu } (x, t) =0, \quad x\in \partial \varOmega , \ t>0, \end{aligned}$$

and that the following initial conditions are satisfied

11 $$\begin{aligned} s_j(x, 0)=s_{j0}(x), \ i_j(x, 0)=i_{j0}(x), \quad&x\in \varOmega , \ j\in \{ 1, 2\} . \end{aligned}$$

**Assumptions**:

- $$s_{10}, \ i_{10}\in L^{\infty }(\varOmega ), \quad s_{10}(x), \ i_{10}(x)\ge 0, \ \mathrm{a.e.} \ x\in \varOmega $$ ;
- $$s_{20}, \ i_{20}\in L^{\infty }(\varOmega ), \quad s_{20}(x), \ i_{20}(x)\ge 0, \ \mathrm{a.e.} \ x\in \varOmega $$ ;
- $$\gamma _1\ge 0$$, $$\gamma _{21}, \ \gamma _{22}, \ \gamma _{23}\in [0,1]$$, $$\gamma _3\in [0,1]$$ are constants.

Let $(s_1,i_1,s_2,i_2)$ be the solution to (6)–(11), satisfying

$$\begin{aligned} s_j, \ i_j\in L^{\infty }_{loc}(\overline{\varOmega }\times [0,+\infty )) , \\ s_j(x,t), \ i_j(x,t)\ge 0, \ \mathrm{a.e.} \ (x,t)\in \varOmega \times (0,+\infty ) , \end{aligned}$$

$j\in \{1,2\}$. We postpone, for the time being, the proof of the existence and uniqueness of such a solution.

As far as the trees are concerned, we may easily obtain an upper bound for the total canopy.

If we now add Eqs. (8) and (9) and take into account the initial conditions, we find that $C_T=s_2+i_2$ satisfies

12 $$\begin{aligned} \left\{ \begin{array}{ll} \displaystyle \frac{\partial C_T}{\partial t} =(q-\ell )C_T-\frac{1}{C}C_T^2-(\mu +q)i_2, &{}\quad x\in \varOmega , \ t>0 \\ C_T(x,0)=C_{T0}(x)=s_{20}(x)+i_{20}(x), &{} \quad x\in \varOmega . \end{array} \right. \end{aligned}$$

If we denote by $\tilde{C}_T$ the solution to

13 $$\begin{aligned} \left\{ \begin{array}{ll} \displaystyle \frac{\mathrm{d} \tilde{C}_T}{\mathrm{d} t} =(q-\ell )\tilde{C}_T-\frac{1}{C}\tilde{C}_T^2, &{} \quad t>0 \\ \tilde{C}_T(0)=\Vert C_{T0}\Vert _{\infty }+1, &{} \end{array} \right. \end{aligned}$$

we may state that

14 $$\begin{aligned} C_T(x,t)\le \tilde{C}_T(t), \quad \mathrm{a.e.} \ (x,t)\in \varOmega \times (0,+\infty ) \end{aligned}$$

(here, and throughout this paper, $\Vert \cdot \Vert _p$ denotes the usual norm in $L^p(\varOmega )$); it is obvious that $\tilde{C}_T$ is space independent.

To prove this inequality, we set $y(x,t):=\tilde{C}_T(t)-C_T(x,t)$ and note that *y* is the solution to

$$\begin{aligned} \left\{ \begin{array}{ll} \displaystyle \frac{\partial y}{\partial t}(x,t)=\theta (x,t)y(x,t)+g(x,t), &{} \quad x\in \varOmega , \ t>0 \\ y(x,0)=y_0(x), &{}\quad x\in \varOmega , \end{array} \right. \end{aligned}$$

where $y_0(x)=\tilde{C}_T(0)-C_T(x,0)$, $\theta (x,t)=q-\ell -\frac{1}{C}(\tilde{C}_T(t)+C_T(x,t))$, $g(x,t)=(\mu +q)i_2(x,t)$, if $x\in \varOmega$, $t>0$. Since $y_0$ and *g* are nonnegative, we have that $y(x,t)\ge 0$, a.e. $x\in \varOmega$, $\forall t\ge 0$, and the inequality (14) follows.

As a consequence of (13)

(j) if $q \le \ell ,$ then $\tilde{C}_T(t)\rightarrow 0$, as $t\rightarrow +\infty ;$ hence, $C_T(x, t)\rightarrow 0$, a.e. $x\in \varOmega ,$ as $t\rightarrow +\infty ;$
(jj) if $q>\ell ,$ then $\tilde{C}_T(t)\rightarrow C(q-\ell )$, as $t\rightarrow +\infty$.

We note that the quantity $C(q-\ell )^+$ is the carrying capacity of the tree population.

We may now also note that if we denote by $\overline{C}_T$ the solution to

$$\begin{aligned} \left\{ \begin{array}{ll} \displaystyle \frac{\mathrm{d} \overline{C}_T}{\mathrm{d}t} =(q-\ell )\overline{C}_T, &{} \quad t>0 \\ \overline{C}_T(0)=\Vert C_{T0}\Vert _{\infty }+1, &{} \end{array} \right. \end{aligned}$$

by similar arguments as for the previous inequality, we may also state that

$$\begin{aligned} \tilde{C}_T(t) \le \overline{C}_T(t), \quad \ t>0. \end{aligned}$$

It is evident that $\overline{C}_T$ is space independent too. Altogether we may state

$$\begin{aligned} C_T(x,t)\le \tilde{C}_T(t)\le \overline{C}_T(t), \quad \mathrm{a.e. } \ (x,t)\in \varOmega \times (0,+\infty ) , \end{aligned}$$

For the insect population, we may add Eqs. (6) and (7) and take into account the boundary and initial conditions, to obtain that $C_I=s_1+i_1$ satisfies

15 $$\begin{aligned} \left\{ \begin{array}{ll} \displaystyle \frac{\partial C_I}{\partial t} =d\varDelta C_I+r[M(x)(1-\gamma _{21}\mathbb I_{\omega }(x))-\chi C_I]C_I-(n+\gamma _1\mathbb I_{\omega }(x))C_I, &{}x\in \varOmega , \ t>0 \\ \displaystyle \frac{\partial C_I}{\partial \nu } (x, t)=0, &{}x\in \partial \varOmega , \ t>0 \\ C_I(x,0)=C_{I0}(x)=s_{10}(x)+i_{10}(x), &{}x\in \varOmega . \end{array} \right. \end{aligned}$$

By Banach’s fixed point theorem and using the comparison result for the solutions to the linear parabolic equations [see Friedman (1964) and Protter and Weinberger (1994)], the existence and uniqueness of a nonnegative solution to (15) follow. Moreover, the solution satisfies

$$\begin{aligned} C_I(x,t)\le \overline{C}_I(x,t), \quad \mathrm{a.e. } \ (x,t)\in \varOmega \times (0,+\infty ) , \end{aligned}$$

where $\overline{C}_I$ is the solution to the linear parabolic equation

$$\begin{aligned} \left\{ \begin{array}{ll} \displaystyle \frac{\partial \overline{C}_I}{\partial t} =d\varDelta \overline{C}_I+rM(x)(1-\gamma _{21}\mathbb I_{\omega }(x))\overline{C}_I-(n+\gamma _1\mathbb I_{\omega }(x))\overline{C}_I, &{}\quad x\in \varOmega , \ t>0 \\ \displaystyle \frac{\partial \overline{C}_I}{\partial \nu } (x, t)=0, &{}\quad x\in \partial \varOmega , \ t>0 \\ \overline{C}_I(x,0)=C_{I0}(x)=s_{10}(x)+i_{10}(x), &{}\quad x\in \varOmega . \end{array} \right. \end{aligned}$$

Turning back to (6)–(11), it follows via Banach’s fixed point theorem (and using the comparison of the solutions to the linear parabolic equations and the comparison of the solutions to the linear first order ODEs) that there exists a unique solution $(s_1,i_1,s_2,i_2)$ such that

$$\begin{aligned}&s_j, \ i_j\in L^{\infty }_{loc}(\overline{\varOmega }\times [0,+\infty )) , \\&0\le s_1(x,t), \ i_1(x,t)\le \overline{C}_I(x,t), \ \mathrm{a.e.} \ (x,t)\in \varOmega \times (0,+\infty ) , \\&0\le s_2(x,t), \ i_2(x,t)\le \overline{C}_T(t), \ \mathrm{a.e.} \ (x,t)\in \varOmega \times (0,+\infty ) , \end{aligned}$$

$j\in \{1,2\}$ [for such an argument see Aniţa and Capasso (2009, 2012) and Aniţa et al. (2009)].

Let $C_I^*$ be the maximal nonnegative solution to

16 $$\begin{aligned} \left\{ \begin{array}{ll} \displaystyle -d\varDelta C_I-[rM(x)(1-\gamma _{21}\mathbb I_{\omega }(x))-(n+\gamma _1\mathbb I_{\omega }(x))]C_I+r\chi C_I^2=0, &{}\quad x\in \varOmega \\ \displaystyle \frac{\partial C_I}{\partial \nu } (x)=0, &{}\quad x\in \partial \varOmega , \end{array} \right. \end{aligned}$$

and let $\tilde{\lambda }_1$ be the principal eigenvalue for the problem

17 $$\begin{aligned} \left\{ \begin{array}{ll} \displaystyle -d\varDelta \varphi -[rM(x)(1-\gamma _{21}\mathbb I_{\omega }(x))-(n+\gamma _1\mathbb I_{\omega }(x))]\varphi =\lambda \varphi , &{}\quad x\in \varOmega \\ \displaystyle \frac{\partial \varphi }{\partial \nu } (x)=0, &{}\quad x\in \partial \varOmega . \end{array} \right. \end{aligned}$$

It is obvious that $\tilde{\lambda }_1$ depends on $\varOmega , \omega$, and the controls $\gamma _1$ and $\gamma _{21}$, and is an increasing function of $\gamma _1,$ $\gamma _{21}$, and $\omega$—by inclusion—via Rayleigh’s principle.

By the same methods as in Aniţa et al. (2009), the following proposition can be shown to hold.

### Proposition 1

Under the above assumptions

(i) If $\tilde{\lambda }_1\ge 0$, then (16) admits only the trivial solution.
  Moreover, for any initial condition, the solution to (15) satisfies
  $$\begin{aligned} C_I(\cdot ,t)\rightarrow 0 \quad \mathrm{in } \ L^{\infty }(\varOmega ), \end{aligned}$$
  as $$t\rightarrow +\infty $$.
(ii) If $\tilde{\lambda }_1<0$, then (16) has two nonnegative solutions: the trivial one and $C_I^* >0.$
  Moreover, if $C_{I0}$ is not identically 0, then the solution to (15) satisfies
  $$\begin{aligned} C_I(\cdot ,t)\rightarrow C_I^* \quad \mathrm{in } \ L^{\infty }(\varOmega ) , \end{aligned}$$
  as $$t\rightarrow +\infty .$$

We note that Proposition 1 confirms for the full reaction–diffusion system, the outcomes of the preliminary analysis presented at the beginning of Sect. 3. In particular, it is of interest to observe that, for small values of the weed distribution, *M*(*x*),  or a large value of the control parameter, $\gamma _{21},$ or a large domain of intervention, $\omega ,$ $\tilde{\lambda }_1$ may become greater than or equal to zero, so that the only nonnegative solution of (16) is the trivial one, and the whole insect population eventually goes extinct.

The case $\tilde{\lambda }_1 < 0$ requires further investigation, since an additional nontrivial value of $C_I^*$ is possible; we may then require suitable threshold conditions for the eventual extinction of the epidemic.

Now, let $\lambda _1$ be the principal eigenvalue for the problem

18 $$\begin{aligned} \left\{ \begin{array}{ll} -d\varDelta \varphi +(n+\gamma _1\mathbb I_{\omega }(x))\varphi +r\chi C_I^*(x)\varphi =\lambda \varphi , &{}\quad x\in \varOmega \\ \displaystyle \frac{\partial \varphi }{\partial \nu }=0, &{}\quad x\in \partial \varOmega . \end{array} \right. \end{aligned}$$

Notice that $\lambda _1\ge n$ and that if $\gamma _1\nearrow +\infty$, then $\lambda _1\nearrow \lambda _1^*$, where $\lambda _1^*$ is the principal eigenvalue for

$$\begin{aligned} \left\{ \begin{array}{ll} -d\varDelta \varphi +n\varphi +r\chi C_I^*(x)\varphi =\lambda \varphi , &{}\quad x\in \varOmega \setminus \overline{\omega }\\ \varphi =0, &{}\quad x\in \partial \omega \\ \displaystyle \frac{\partial \varphi }{\partial \nu }=0, &{}\quad x\in \partial \varOmega . \end{array} \right. \end{aligned}$$

Notice that $\lambda _1^*$ may be as large as we wish if $\gamma _1$ is sufficiently large and/or if $\omega$ is sufficiently large.

The following result concerns the eradicability of the disease in the most interesting situation when $\tilde{\lambda }_1<0, q>\ell$ and $C_{I0}\ne 0_{L^{\infty }(\varOmega )}$.

### Theorem 1

If $$\tilde{\lambda }_1<0, q>\ell $$, $$C_{I0}\ne 0_{L^{\infty }(\varOmega )}$$ and

19 $$\begin{aligned} {[}\Vert C_I^*\Vert _{\infty }\Vert k\Vert _2+\zeta C(q-\ell )^+]^2 < \lambda _1[\mu +l+\alpha -b\ell C(q-\ell )^+], \end{aligned}$$

then

$$\begin{aligned} i_1(\cdot ,t)\longrightarrow 0, \quad i_2(\cdot ,t)\longrightarrow 0 \quad \mathrm{in } \ L^1(\varOmega ), \end{aligned}$$

as $$t\rightarrow +\infty $$.

Notice that condition (19) holds if, for example, $\gamma _1$ is sufficiently large and/or the subset $\omega$ is sufficiently large.

### Proof

Since $$C_{I0}\ne 0_{L^{\infty }(\varOmega )}$$, then $$\forall \varepsilon >0, \exists t(\varepsilon )\ge 0, \forall t, t\ge t(\varepsilon ):$$

$$\begin{aligned} C_I^*(x)-\varepsilon< C_I(x,t) < C_I^*(x) +\varepsilon , \quad \mathrm{a.e. } \ x\in \varOmega , \ t\ge t(\varepsilon ), \end{aligned}$$

and

$$\begin{aligned} 0\le C_T(x,t) <C(q-\ell )^++\varepsilon , \quad \mathrm{a.e. } \ x\in \varOmega , \ t\ge t(\varepsilon ). \end{aligned}$$

Let $(\tilde{\i }_1,\tilde{\i }_2)$ be the solution to

20 $$\begin{aligned} \left\{ \begin{array}{ll} \displaystyle \frac{\partial \tilde{\i }_1}{\partial t} =d\varDelta \tilde{\i }_1-(n+\gamma _1\mathbb I_{\omega }(x))\tilde{\i }_1-r\chi (C_I^*(x)-\varepsilon )\tilde{\i }_1 &{} \\ \\ ~~~~~~~~~\quad +\,\displaystyle (C_I^*(x)+\varepsilon )(1-\gamma _{23}\mathbb I_{\omega }(x))\int _{\varOmega }k(x,x')\tilde{\i }_2(x',t)\mathrm{d}x', &{}x\in \varOmega , \ t>t(\varepsilon ) \\ \\ \displaystyle \frac{\partial \tilde{\i }_1}{\partial \nu } (x, t)=0, &{} x\in \partial \varOmega , \ t>t(\varepsilon ) \\ \\ \displaystyle \frac{\partial \tilde{\i }_2}{\partial t}=-(\mu +\ell )\tilde{\i }_2 -\alpha \tilde{\i }_2 &{} \\ ~~~~~~~~\quad +\,[\zeta (1-\gamma _{22}\mathbb I_{\omega }(x))\tilde{\i }_1+ b(1 - \gamma _3\mathbb I_{\omega }(x))\ell \tilde{\i }_2]\cdot [C(q-\ell )^++\varepsilon ], &{}x\in \varOmega , \ t>t(\varepsilon ) \\ \\ \tilde{\i }_1(x,t(\varepsilon ))=i_1(x,t(\varepsilon )), \ \tilde{\i }_2(x,t(\varepsilon ))=i_2(x,t(\varepsilon )), &{}x\in \varOmega \end{array} \right. \end{aligned}$$

(the existence, uniqueness, and nonnegativity of the solution follows via a fixed point argument). We have that

$$\begin{aligned} 0\le i_j(x,t)\le \tilde{\i }_j(x,t) \quad \mathrm{a.e. } \ x\in \varOmega , \forall t\ge t(\varepsilon ), \end{aligned}$$

$j\in \{ 1,2\}$. This follows from considering the system satisfied by $(\tilde{\i }_1-i_1,\tilde{\i }_{2}-i_2)$ and showing in a standard manner that

$$\begin{aligned} (\tilde{\i }_1-i_1)^-(x,t)=0, \ (\tilde{\i }_1-i_1)^-(x,t), \ x\in \varOmega , \ t\ge t(\varepsilon ). \end{aligned}$$

If we multiply the first equation in (20) by $\tilde{\i }_1$ and integrate over $\varOmega$, we obtain that

$$\begin{aligned} \frac{1}{2}\frac{\mathrm{d}}{\mathrm{d}t}\left( \int _{\varOmega }|\tilde{\i }_1(x,t)|^2\mathrm{d}x\right)\le & {} -d\int _{\varOmega }|\nabla \tilde{\i }_1(x,t)|^2\mathrm{d}x-\int _{\varOmega }(n+\gamma _1\mathbb I_{\omega }(x))|\tilde{\i }_1(x,t)|^2\mathrm{d}x \\&-\, r\chi \int _{\varOmega }(C_I^*(x)-\varepsilon )|\tilde{\i }_1(x,t)|^2\mathrm{d}x \\&+\,\int _{\varOmega }(C_I^*(x)+\varepsilon )\tilde{\i }_1(x,t)\int _{\varOmega }k(x,x')\tilde{\i }_2(x',t)\mathrm{d}x'\mathrm{d}x \\\le & {} -\,\lambda _1\Vert \tilde{\i }_1(t)\Vert _2^2+r\chi \varepsilon \Vert \tilde{\i }_1(t)\Vert _2^2+(\Vert C_I^*\Vert _{\infty }+\varepsilon )\Vert k\Vert _2 \Vert \tilde{\i }_1(t)\Vert _2 \Vert \tilde{\i }_2(t)\Vert _2 \end{aligned}$$

(by Rayleigh’s principle), $\forall t\ge t(\varepsilon ).$

If we multiply the second PDE in (20) by $\tilde{\i }_2$ and integrate over $\varOmega$, we obtain that

$$\begin{aligned} \frac{1}{2}\frac{\mathrm{d}}{\mathrm{d}t}\left( \int _{\varOmega }|\tilde{\i }_2(x,t)|^2\mathrm{d}x\right)= & {} -(\mu +\ell )\int _{\varOmega }|\tilde{\i }_2(x,t)|^2\mathrm{d}x \\&+\,b\ell [C(q-\ell )^++\varepsilon ]\int _{\varOmega }|\tilde{\i }_2(x,t)|^2\mathrm{d}x-\alpha \int _{\varOmega } |\tilde{\i }_2(x,t)|^2\mathrm{d}x \\&+\,\zeta [C(q-\ell )^++\varepsilon ]\int _{\varOmega }\tilde{\i }_1(x,t)\tilde{\i }_2(x,t)\mathrm{d}x \\\le & {} -\,[\mu +\ell +\alpha -bl(C(q-\ell )^++\varepsilon )]\Vert \tilde{\i }_2(t)\Vert _2^2 \\&+\,\zeta [C(q-\ell )^++\varepsilon ]\Vert \tilde{\i }_1(t)\Vert _2\Vert \tilde{\i }_2(t)\Vert _2, \quad \forall t\ge t(\varepsilon ). \end{aligned}$$

We may infer that

$$\begin{aligned} \frac{1}{2}\frac{\mathrm{d}}{\mathrm{d}t}(\Vert \tilde{\i }_1(t)\Vert _2^2+\Vert \tilde{\i }_2(t)\Vert _2^2)\le & {} -(\lambda _1-r\chi \varepsilon )\Vert \tilde{\i }_1(t)\Vert _2^2 \\&-\,[\mu +\ell +\alpha - b\ell (C(q-\ell )^++\varepsilon )]\Vert \tilde{\i }_2(t)\Vert _2^2 \\&+\,[(\Vert C_I^*\Vert _{\infty }+\varepsilon )\Vert k\Vert _2+\zeta (C(q-\ell )^++\varepsilon )]\Vert \tilde{\i }_1(t)\Vert _2\Vert \tilde{\i }_2(t)\Vert _2 \\\le & {} -\,\frac{1}{2}\min \{ \lambda _1-r\chi \varepsilon , \mu +\ell +\alpha - b\ell (C(q-\ell )^++\varepsilon )\} (\Vert \tilde{\i }_1(t)\Vert _2^2+\Vert \tilde{\i }_2(t)\Vert _2^2), \end{aligned}$$

$$\forall t\ge t(\varepsilon )$$, if

$$\begin{aligned}&{[}(\Vert C_I^*\Vert _{\infty }+\varepsilon )\Vert k\Vert _2+\zeta (C(q-\ell )^++\varepsilon )]^2 \\&\quad < (\lambda _1-r\chi \varepsilon )[\mu +\ell +\alpha -b\ell (C(q-\ell )^++\varepsilon )]. \end{aligned}$$

This condition holds for any sufficiently small $\varepsilon >0$ [because (19) holds]. On the other hand, since condition (19) holds, it follows that for any $\varepsilon >0$ sufficiently small, we have that

$$\begin{aligned} \frac{1}{2}\min \{ \lambda _1-r\chi \varepsilon , \mu +\ell +\alpha - b\ell (C(q-\ell )^++\varepsilon )\} =a>0, \end{aligned}$$

and we conclude that $\Vert \tilde{\i }_1(t)\Vert ^2_2+\Vert \tilde{\i }_2(t)\Vert ^2_2$ converges to 0 as $t\rightarrow +\infty$, at the rate of $\exp \{-2at\}$. Since $\varOmega$ is bounded, it follows that

$$\begin{aligned} \tilde{\i }_1(\cdot ,t) \rightarrow 0, \quad \tilde{\i }_2(\cdot ,t) \rightarrow 0 \quad \mathrm{in } \ L^1(\varOmega ), \end{aligned}$$

and that

$$\begin{aligned} i_1(\cdot ,t) \rightarrow 0, \quad i_2(\cdot ,t) \rightarrow 0 \quad \mathrm{in } \ L^1(\varOmega ), \end{aligned}$$

as $t\rightarrow +\infty$, at least as fast as $\exp \{-at\}$ (i.e., the total number of infected insects and the total number of infected trees tend to 0, exponentially). It means that the disease is eradicable. $\square$

### Remark 1

Recall that if the diffusion coefficient is strictly positive, $d>0,$ then any point of the domain $\varOmega$ is strongly connected to any other point of $\varOmega ,$ so that actions taken in a subregion $\omega \subset \varOmega$ will eventually propagate to the whole habitat.

On the other hand, due to the lack of diffusion for $i_2,$ it is reasonable to expect the control to be more effective (leading to a faster decay of $i_1$ and $i_2$) if $\gamma _{22}, \gamma _{23},$ and $\gamma _3$ act on the whole domain $\varOmega .$ In this case, the controlled system becomes

21 $$\begin{aligned} \frac{\partial s_1}{\partial t} (x, t)&= d\varDelta s_1(x, t) + r C_I(x, t) [ M(x)(1 - \gamma _{21}\mathbb I_{\omega }(x)) - \chi s_1(x, t)] \nonumber \\&\quad - (n + \gamma _1\mathbb I_{\omega }(x)) s_1(x, t) - \beta (1 - \gamma _{23}) s_1(x, t) \displaystyle \int _{\varOmega }k(x,x^{\prime })i_{2}(x^{\prime },t)\mathrm{d}x^{\prime }, \end{aligned}$$

22 $$\begin{aligned} \frac{\partial i_1}{\partial t}(x,t)&=d\varDelta i_{1}(x,t)\displaystyle - (n + \gamma _1\mathbb I_{\omega }(x)) i_{1}(x,t) - r \chi i_{1}(x,t) C_I(x, t) \nonumber \\&\quad + \beta (1 - \gamma _{23}) s_1(x, t) \displaystyle \int _{\varOmega }k(x,x^{\prime })i_{2}(x^{\prime },t)\mathrm{d}x^{\prime }, \end{aligned}$$

23 $$\begin{aligned} \frac{\partial s_2}{\partial t} (x, t)&=(q-\ell ) s_2(x, t) - s_2(x, t) \displaystyle \frac{C_T(x, t)}{C} \nonumber \\&\quad - (\zeta (1- \gamma _{22}) i_1(x, t) + b(1 - \gamma _3) \ell i_2(x, t)) s_2(x, t) + \alpha i_2(x, t), \end{aligned}$$

24 $$\begin{aligned} \frac{\partial i_2}{\partial t} (x, t)&= - \mu i_2(x, t) - \ell i_2 (x, t)- i_2(x, t) \displaystyle \frac{C_T(x, t)}{C} \nonumber \\&\quad + (\zeta (1- \gamma _{22}) i_1(x, t) + b(1 - \gamma _3) \ell i_2(x, t)) s_2(x, t) - \alpha i_2(x, t). \end{aligned}$$

The boundary conditions and the initial conditions are as before.

There exists a unique solution $(s_1,i_1,s_2,i_2)$ to (21)–(24) with the above mentioned boundary, and initial conditions, satisfying

$$\begin{aligned}&s_j, \ i_j\in L^{\infty }_{loc}(\overline{\varOmega }\times [0,+\infty )) , \\&s_j(x,t), \ i_j(x,t)\ge 0, \ \mathrm{a.e.} \ (x,t)\in \varOmega \times (0,+\infty ) , \end{aligned}$$

$j\in \{1,2\}$. This follows from using a similar argument as for (6)–(11).

In this special case, the following result holds:

### Theorem 2

If $$\tilde{\lambda }_1<0, q>\ell $$, $$C_{I0}\ne 0_{L^{\infty }(\varOmega )}$$ and

25 $$\begin{aligned}&{[}(1-\gamma _{23})\Vert C_I^*\Vert _{\infty }\Vert k\Vert _2+\zeta (1-\gamma _{22})C(q-\ell )^+]^2 \nonumber \\&\quad < \lambda _1[\mu +l+\alpha -b(1-\gamma _3)\ell C(q-\ell )^+] \end{aligned}$$

then

$$\begin{aligned} i_1(\cdot ,t)\longrightarrow 0, \quad i_2(\cdot ,t)\longrightarrow 0 \quad \mathrm{in } \ L^1(\varOmega ), \end{aligned}$$

as $$t\rightarrow +\infty $$.

Notice that (25) is a weaker assumption than (19).

### Proof

Using a comparison result for $(i_1,i_2)$, we obtain that

$$\begin{aligned} 0\le i_j(x,t)\le \tilde{\i }_j(x,t) \quad \mathrm{a.e.} \ x\in \varOmega , \forall t\ge t(\varepsilon ), \end{aligned}$$

$j\in \{ 1,2\}$, where $(\tilde{\i }_1,\tilde{\i }_2)$ is the solution to

26 $$\begin{aligned} \left\{ \begin{array}{ll} \displaystyle \frac{\partial \tilde{\i }_1}{\partial t} =d\varDelta \tilde{\i }_1-(n+\gamma _1\mathbb I_{\omega }(x))\tilde{\i }_1-r\chi (C_I^*(x)-\varepsilon )\tilde{\i }_1 &{} \\ \\ ~~~\quad \quad +\,\displaystyle (C_I^*(x)+\varepsilon )(1-\gamma _{23})\int _{\varOmega }k(x,x')\tilde{\i }_2(x',t)\mathrm{d}x', &{} x\in \varOmega , \ t>t(\varepsilon ) \\ \\ \displaystyle \frac{\partial \tilde{\i }_1}{\partial \nu } (x, t)=0, &{} x\in \partial \varOmega , \ t>t(\varepsilon ) \\ \\ \displaystyle \frac{\partial \tilde{\i }_2}{\partial t}=-(\mu +\ell )\tilde{\i }_2 -\alpha \tilde{\i }_2 &{} \\ ~~~\quad \quad +\,[\zeta (1-\gamma _{22})\tilde{\i }_1+ b(1 - \gamma _3)\ell \tilde{\i }_2]\cdot [C(q-\ell )^++\varepsilon ], &{}x\in \varOmega , \ t>t(\varepsilon ) \\ \\ \tilde{\i }_1(x,t(\varepsilon ))=i_1(x,t(\varepsilon )), \ \tilde{\i }_2(x,t(\varepsilon ))=i_2(x,t(\varepsilon )), &{}x\in \varOmega . \end{array} \right. \end{aligned}$$

If we multiply the first equation in (26) by $\tilde{\i }_1$ and integrate over $\varOmega$, we obtain that

$$\begin{aligned} \frac{1}{2}\frac{\mathrm{d}}{\mathrm{d}t}\left( \int _{\varOmega }|\tilde{\i }_1(x,t)|^2\mathrm{d}x\right)\le & {} -d\int _{\varOmega }|\nabla \tilde{\i }_1(x,t)|^2\mathrm{d}x-\int _{\varOmega }(n+\gamma _1\mathbb I_{\omega }(x))|\tilde{\i }_1(x,t)|^2\mathrm{d}x \\&-r\chi \int _{\varOmega }(C_I^*(x)-\varepsilon )|\tilde{\i }_1(x,t)|^2\mathrm{d}x \\&+(1-\gamma _{23})\int _{\varOmega }(C_I^*(x)+\varepsilon )\tilde{\i }_1(x,t)\int _{\varOmega }k(x,x')\tilde{\i }_2(x',t)\mathrm{d}x'\mathrm{d}x \\\le & {} -\lambda _1\Vert \tilde{\i }_1(t)\Vert _2^2+r\chi \varepsilon \Vert \tilde{\i }_1(t)\Vert _2^2\\&+(1-\gamma _{23})(\Vert C_I^*\Vert _{\infty }+\varepsilon )\Vert k\Vert _2 \Vert \tilde{\i }_1(t)\Vert _2 \Vert \tilde{\i }_2(t)\Vert _2 \end{aligned}$$

(by Rayleigh’s principle).

If we multiply the second PDE in (26) by $\tilde{\i }_2$ and integrate over $\varOmega$, we obtain that

$$\begin{aligned} \frac{1}{2}\frac{\mathrm{d}}{\mathrm{d}t}\left( \int _{\varOmega }|\tilde{\i }_2(x,t)|^2\mathrm{d}x\right)= & {} -(\mu +\ell )\int _{\varOmega }|\tilde{\i }_2(x,t)|^2\mathrm{d}x \\&+\,b(1-\gamma _3)\ell [C(q-\ell )^++\varepsilon ]\int _{\varOmega }|\tilde{\i }_2(x,t)|^2\mathrm{d}x\\&-\alpha \int _{\varOmega } |\tilde{\i }_2(x,t)|^2\mathrm{d}x \\&+\,\zeta (1-\gamma _{22})[C(q-\ell )^++\varepsilon ]\int _{\varOmega }\tilde{\i }_1(x,t)\tilde{\i }_2(x,t)\mathrm{d}x \\\le & {} -\,[\mu +\ell +\alpha -b(1-\gamma _3))l(C(q-\ell )^++\varepsilon )]\Vert \tilde{\i }_2(t)\Vert _2^2 \\&+\,\zeta (1-\gamma _{22})[C(q-\ell )^++\varepsilon ]\Vert \tilde{\i }_1(t)\Vert _2\Vert \tilde{\i }_2(t)\Vert _2, \quad \forall t\ge t(\varepsilon ). \end{aligned}$$

We may infer that

$$\begin{aligned} \frac{1}{2}\frac{\mathrm{d}}{\mathrm{d}t}(\Vert \tilde{\i }_1(t)\Vert _2^2+\Vert \tilde{\i }_2(t)\Vert _2^2)\le & {} -(\lambda _1-r\chi \varepsilon )\Vert \tilde{\i }_1(t)\Vert _2^2 \\&-\,[\mu +\ell +\alpha - b(1-\gamma _3)\ell (C(q-\ell )^++\varepsilon )]\Vert \tilde{\i }_2(t)\Vert _2^2 \\&+\,[(1-\gamma _{23})(\Vert C_I^*\Vert _{\infty }+\varepsilon )\Vert k\Vert _2+\zeta (1-\gamma _{22})(C(q-\ell )^++\varepsilon )]\\&\times \Vert \tilde{\i }_1(t)\Vert _2\Vert \tilde{\i }_2(t)\Vert _2 \\\le & {} -\,\frac{1}{2}\min \{ \lambda _1-r\chi \varepsilon , \mu +\ell +\alpha - b(1-\gamma _3)\ell (C(q-\ell )^++\varepsilon )\}\\&\times (\Vert \tilde{\i }_1(t)\Vert _2^2+\Vert \tilde{\i }_2(t)\Vert _2^2), \end{aligned}$$

$$\forall t\ge t(\varepsilon )$$, if

$$\begin{aligned}&{[}((1-\gamma _{23})\Vert C_I^*\Vert _{\infty }+\varepsilon )\Vert k\Vert _2+\zeta (1-\gamma _{22})(C(q-\ell )^++\varepsilon )]^2 \\&\quad < (\lambda _1-r\chi \varepsilon )[\mu +\ell +\alpha -b(1-\gamma _3)\ell (C(q-\ell )^++\varepsilon )]. \end{aligned}$$

This condition holds for $\varepsilon >0$ sufficiently small (because (25) holds). On the other hand, since condition (25) is satisfied, then for $\varepsilon >0$ sufficiently small, we have that

$$\begin{aligned} \frac{1}{2}\min \{ \lambda _1-r\chi \varepsilon , \mu +\ell +\alpha - b(1-\gamma _3)\ell (C(q-\ell )^++\varepsilon )\} =\tilde{a}>0, \end{aligned}$$

and we conclude that $\Vert \tilde{\i }_1(t)\Vert ^2_2+\Vert \tilde{\i }_2(t)\Vert ^2_2$ converges to 0 as $t\rightarrow +\infty$, at the rate of $\exp \{-2\tilde{a}t\}$. Since $\varOmega$ is bounded, it follows that

$$\begin{aligned} \tilde{\i }_1(\cdot ,t) \rightarrow 0, \quad \tilde{\i }_2(\cdot ,t) \rightarrow 0 \quad \mathrm{in } \ L^1(\varOmega ), \end{aligned}$$

and that

$$\begin{aligned} i_1(\cdot ,t) \rightarrow 0, \quad i_2(\cdot ,t) \rightarrow 0 \quad \mathrm{in } \ L^1(\varOmega ), \end{aligned}$$

as $t\rightarrow +\infty$, at least as fast as $\exp \{-\tilde{a}t\}$ (i.e., the total number of infected insects and the total number of infected trees tend to 0, exponentially). This means that the disease is eradicable. Notice that $\tilde{a}\ge a$. $\square$

## Numerical Simulations

The numerical strategy adopted to approximate the controlled system (Equations (6)–(9)) consists of the finite element method for space discretization and the finite difference method for time discretization. This procedure is the state of the art for the solution of parabolic partial differential equations (PDEs); see, e.g., Quarteroni and Valli (1994).

*Space discretization* We first apply a standard Galerkin procedure to the weak formulations of the controlled system [Eqs. (6)–(9)]. For the computational domain $\varOmega$, we have taken a rectangle of size $400\times 80\,\mathrm{km}^2,$ which mimics, for example, the whole region of Apulia in Southern Italy, from South (right-hand side of the domain) to North (left-hand side of the domain). The rectangular domain has been discretized by a uniform grid of $200\times 40$ bilinear finite elements (*Q*1), yielding a total number of 8241 discretization nodes. The stiffness matrix is computed exactly, whereas the mass matrix is obtained by applying the mass lumping technique.

*Time discretization* After the spatial discretization, we obtain a semi-discrete problem that consists of a system of ordinary differential equations (ODEs). We solve these ODEs by employing a first order semi-implicit finite difference scheme, where the linear diffusion terms are approximated by Backward Euler, whereas the nonlinear reaction terms are approximated by forward Euler. As a result, at each time step we must find the solution of a linear system of algebraic equations of dimension $4\times 8241=32964$ degrees of freedom. The system is solved by Gaussian elimination with the built-in function in MATLAB. The time step size is $\varDelta t=0.0002\,\mathrm{years}$. For further details on the numerical discretization of parabolic problems, we refer the reader to Quarteroni and Valli (1994).

### Parameter Calibration

**Table 2:** Values of the model parameters

Symbol	Unit	Value range	Simulation value	References
*r*	year$^{-1}$	[37,400]	200	Silva et al. (2015) and Yurtsever (2000)
$\chi$	–	[0.001, 0.05]	0.03	Brunetti et al. (2020)
*n*	year$^{-1}$	[0.95, 0.99]	0.98	Brunetti et al. (2020)
*q*	year$^{-1}$	[0.2, 0.7]	0.5	Villalobos et al. (2006)
*C*	–	100	100	Brunetti et al. (2020)
$\ell$	year$^{-1}$	0.01	0.01	Brunetti et al. (2020)
*b*	–	0.05	0.05	Brunetti et al. (2020)
$\mu$	year$^{-1}$	[0.8, 1]	0.9	Saponari et al. (2017, 2018)
$\alpha$	year$^{-1}$	[0.1, 0.5]	0.3	Brunetti et al. (2020)
$\beta$	year$^{-1}$	[0.5, 1]	1	Cornara et al. (2017)
$\zeta$	year$^{-1}$	[0.2, 0.8]	0.8	Boscia et al. (2017) and Fierro et al. (2019)

The values of the simulation parameters are listed in Table 2. The diffusion coefficient in the PDEs is set to $d=1e-4\,\mathrm{km}^2/\mathrm{year}$. The total simulation time is $T=10\, \mathrm{years}$. The initial distributions, used in all next numerical simulations, are the following:

$$\begin{aligned} \left\{ \begin{array}{l} s_1(x,0)=100\\ i_1(x,0)=20\exp (-100(x_1-380)^2-100(x_2-40)^2)\\ s_2(x,0)=50\\ i_2(x,0)=0 \end{array} \right. \end{aligned}$$

That is, we assume constant initial distributions for healthy insects and trees, and we localize the initial presence of infected insects on the right-hand side of the domain.

### Numerical Results

A couple of numerical experiments are reported here to show the efficacy of the controls on the weed biomass and the olive cultivar.

**Fig. 1:**
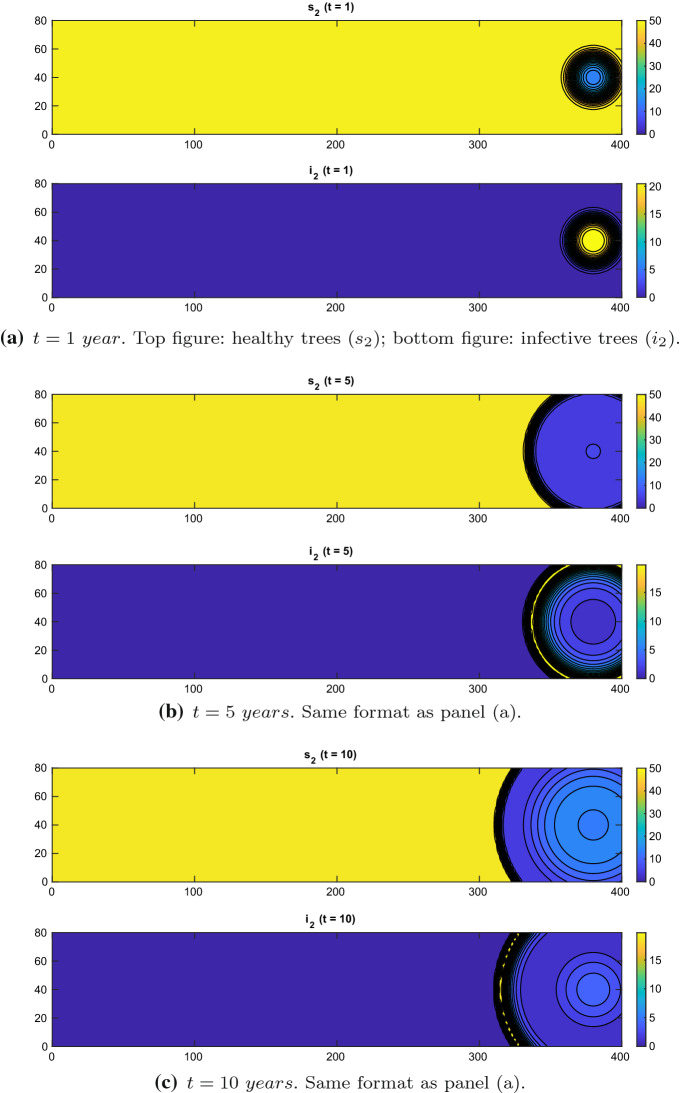
Experiment 1. Spatial distributions of olive trees without control: $\chi = 0.03$, $d=1\mathrm{e}{-}4\,\mathrm{km}^2/\mathrm{year}$ (Color figure online)

**Fig. 2:**
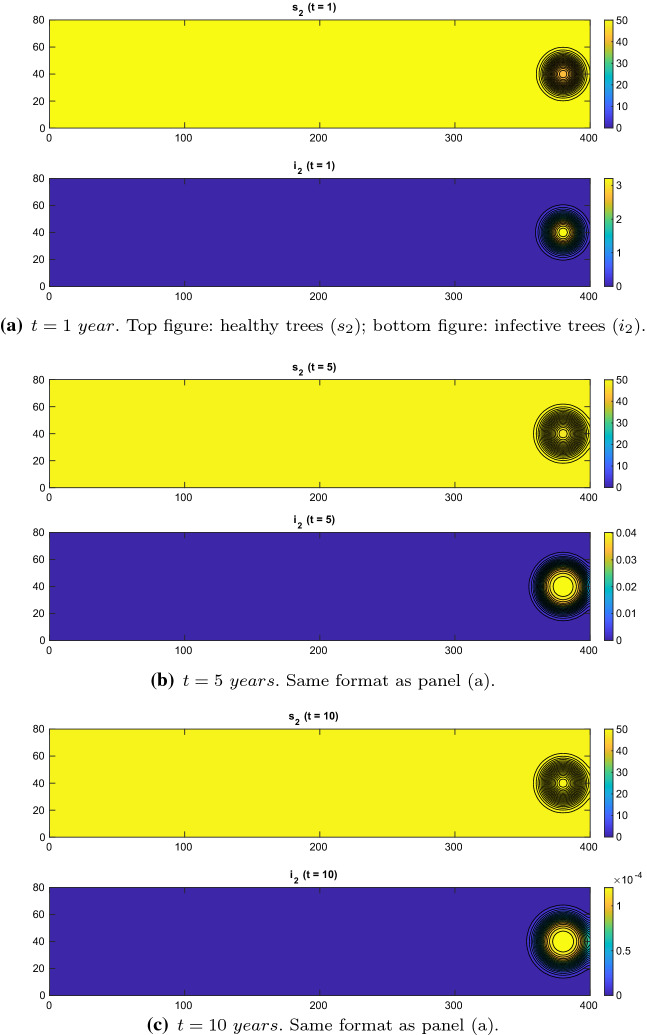
Experiment 1. Spatial distributions of olive trees with regional control. $\chi = 0.03$, $d=1e-4\,\mathrm{km}^2/\mathrm{year}$, $\{x=(x_1,x_2)\in \varOmega :\ x_1>250\,\mathrm{km}\}$. $(\gamma _{21},\gamma _1,\gamma _{23},\gamma _{22},\gamma _3)=(0.999,0.2,0.2,0.2,0.2)$ (Color figure online)

**Fig. 3:**
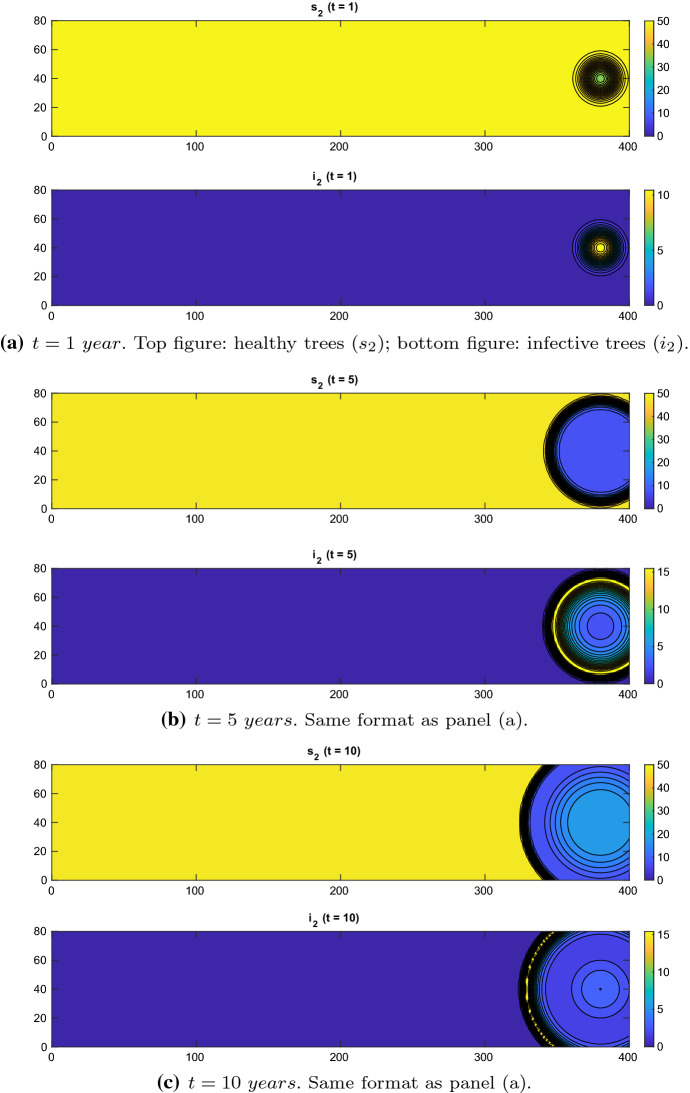
Experiment 2. Spatial distributions of olive trees without control: $\chi = 0.01$, $d=1e-4\,\mathrm{km}^2/\mathrm{year}$ (Color figure online)

**Fig. 4:**
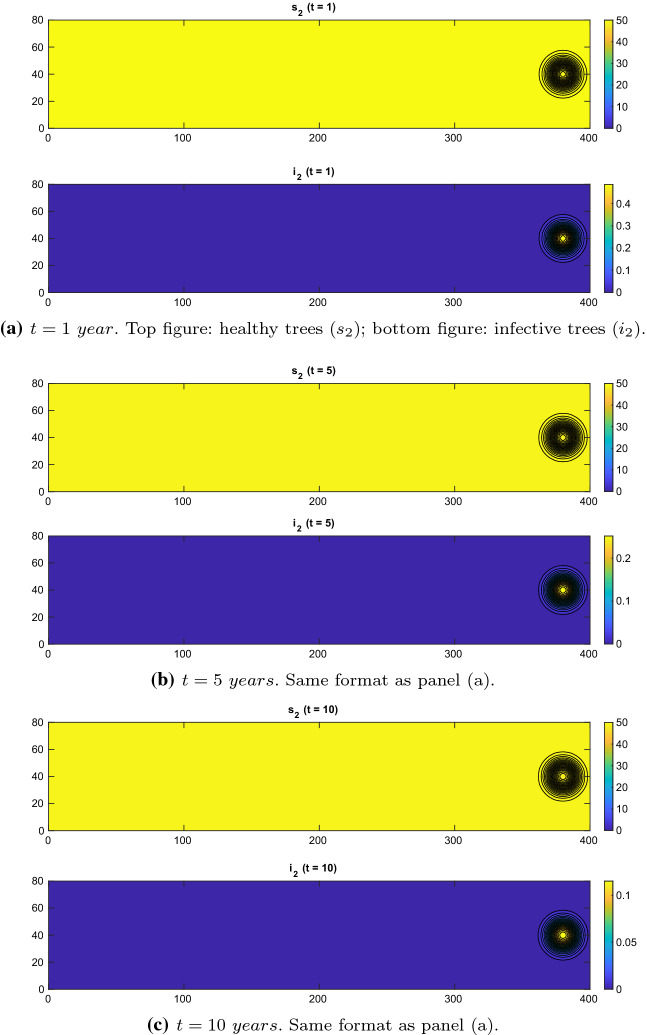
Experiment 2. Spatial distributions of olive trees with regional control. $\chi = 0.01$, $d=1e-4\,\mathrm{km}^2/\mathrm{year}$, $\{x=(x_1,x_2)\in \varOmega :\ x_1>250\,\mathrm{km}\}$. $(\gamma _{21},\gamma _1,\gamma _{23},\gamma _{22},\gamma _3)=(0.6,0.2,0.2,0.6,0.2)$ (Color figure online)

In Figs. 1 and 2, we have taken $M= 1$ and $\chi = 0.03$, while in Figs. 3 and 4, $M= 1$ and $\chi = 0.01$. The colorbar indicates the scaled values of the distribution corresponding to the colors adopted in the map: It goes from blue, associated with low values, to yellow, associated with high values.

In both computational experiments, control measures have been applied only in the right-hand section of the habitat, simulating the subregion of the Apulian region already affected by the xylella epidemic, augmented by a “containment band,” i.e., the subregion

$$\begin{aligned} \omega = \{x=(x_1,x_2)\in \varOmega :\ x_1>250\,\mathrm{km}\}. \end{aligned}$$

*Experiment 1* $\chi = 0.03, \zeta =0.8\,\mathrm{year}^{-1}$: we first run the model without control, and thus, the control parameters ($\gamma _{21}$, $\gamma _1$, $\gamma _{23}$, $\gamma _{22}$, $\gamma _3$) are set to zero everywhere. The xylella epidemic starts spreading as a travelling wave from the right portion of the domain, where the initial condition for the infected insects is positive; see Fig. 1.

When we apply regional control, setting the control parameters

$$\begin{aligned} (\gamma _{21},\gamma _1,\gamma _{23},\gamma _{22},\gamma _3)=(0.999,0.2,0.2,0.2,0.2), \end{aligned}$$

the xylella epidemic is stopped at the origin and no travelling front occurs; see Fig. 2. This result confirms our conjecture that a significant weed cut in the olive orchards strongly decreases the carrying capacity of insects, yielding a successful blocking of the xylella epidemic spread.

In order to investigate how the diffusion coefficient *d* influences the results, we have re-run both tests with $d=2\mathrm{e}{-}4,\ 5\mathrm{e}{-}4,\ 1\mathrm{e}{-}3$ (all in $\mathrm{km}^2/\mathrm{year}$). We do not observe any significant qualitative difference.

*Experiment 2.* $\chi = 0.01, \zeta = 0.2\ year^{-1}:$ as above, we first run the model without control, and thus, the control parameters ($\gamma _{21}$, $\gamma _1$, $\gamma _{23}$, $\gamma _{22}$, $\gamma _3$) are set to zero everywhere. The xylella epidemic starts spreading as a travelling wave from the right portion of the domain, where the initial condition of the infected insects is positive; see Fig. 3.

When we apply a regional control, setting the control parameters

$$\begin{aligned} (\gamma _{21},\gamma _1,\gamma _{23},\gamma _{22},\gamma _3)=(0.6,0.2,0.2,0.6,0.2), \end{aligned}$$

the xylella epidemic is stopped at the origin and no travelling front occurs; see Fig. 4. This result clearly indicates that for more resistant olive cultivar, we may still block an epidemic with a lower level of weed cut in the olive orchards, even though, by using $\chi = 0.001,$ we have a larger effective insect carrying capacity.

As in the previous experiment, we have also re-run both tests with $d=2\mathrm{e}{-}4,\ 5\mathrm{e}{-}4,\ 1\mathrm{e}{-}3$ (all in $\mathrm{km}^2/\mathrm{year}$). Even in this case, we do not observe any significant qualitative difference.

## Concluding Remarks

The results reported in this paper confirm that the most promising target for an effective and cost-efficient control of the *X. fastidiosa* epidemic is represented by agricultural management practices consisting of the removal of the weeds in the whole relevant habitat of the olive orchards. A further interesting strategy, as expected, is represented by the use of more resistant cultivar (Brunetti et al. 2020) [see also Schneider et al. (2020) and references cited therein].

Here, we have extended the ordinary differential equation (ODE) model introduced in Brunetti et al. (2020) to an integro-partial differential system which takes into account the spatial structure of the relevant epidemic system, subject to possible control strategies, including weed cut, insect traps, treated nets, more resistant cultivar, etc. As emphasized in Remark 1, theoretical results show that eradication of an epidemic outbreak is possible by the implementation of the above mentioned control measures only on a suitable subregion of the whole habitat, including the area already affected by the outbreak, possibly augmented by a suitable “containment band.”

Unfortunately in practice, it might be unfeasible to act on the diffusion parameters; they are related to the ecological structure of the relevant habitat, including the behavior of the insect population. Intervention strategies should then include possible modification of insect behavior, such as by the use of treated nets.

Future investigations will be devoted to the search of an optimal set of control parameters, $\gamma$’s, together with an optimal subregion of intervention $\omega .$ It is clear that, for a realistic optimal control problem, relevant participating costs need to be included: e.g., production, losses, and management costs.

Once again, validation of the model proposed here represents a key issue: although we have tried to make explicit the assumptions underlying our model, they have not yet been validated by comparison with experimental data. Therefore, we caution that our results are far from being conclusive for *X. fastidiosa* subsp. *pauca - Philaenus spumarius* olive tree epidemics. However, it is desirable that, with additional features that make it more realistic, our model might provide the foundations for designing optimal control strategies by public authorities.

It is worth reporting here a statement (abridged) taken from the very recent paper (Matricardi et al. 2020) on COVID-19 modeling, which can be applied to the role of any model:

“a model is only an approximate interpretation of reality and it is always wrong in some small or relevant elements. The destiny of the model presented here is to be rapidly improved thanks to novel knowledge coming from new observations and better assumptions. The Authors hope that many and more brilliant minds will read the present pages, will identify and highlight putative mistakes, will get inspiration for their research, and will produce better, more complete, and useful models........ If the speculations presented here on implications for surveillance, control, and therapy of [Xylella] will contribute, even only minimally, to save ..... [olive trees]........., then the Authors have accomplished their small mission. ”
